# Major effect genes or loose confederations? The development of insecticide resistance in the malaria vector *Anopheles gambiae*

**DOI:** 10.1186/1756-3305-3-74

**Published:** 2010-08-17

**Authors:** Basil D Brooke, Lizette L Koekemoer

**Affiliations:** 1 Malaria Entomology Research Unit, School of Pathology of the University of the Witwatersrand and the National Health Laboratory Service, Johannesburg, South Africa; 2 Vector Control Reference Unit, National Institute for Communicable Diseases, NHLS, Private Bag X4, Sandringham, 2131, South Africa

## Abstract

Insecticide use in public health and agriculture presents a dramatic adaptive challenge to target and non-target insect populations. The rapid development of genetically modulated resistance to insecticides is postulated to develop in two distinct ways: By selection for single major effect genes or by selection for loose confederations in which several factors, not normally associated with each other, inadvertently combine their effects to produce resistance phenotypes. Insecticide resistance is a common occurrence and has been intensively studied in the major malaria vector *Anopheles gambiae*, providing a useful model for examining how insecticide resistance develops and what pleiotropic effects are likely to emerge as a consequence of resistance. As malaria vector control becomes increasingly reliant on successfully managing insecticide resistance, the characterisation of resistance mechanisms and their pleiotropic effects becomes increasingly important.

## Introduction

The occurrence of insecticide resistance in insect disease vectors and agricultural pest species poses potential and actual hindrances to successful insect control. Insecticide resistance mechanisms are biological attributes under direct genetic control, and a fundamental issue arising with the development of resistance is the mode and number of genetic factors that translate into resistant phenotypes.

The key caveat imposed on individual insects and on insect populations under insecticide pressure is the production of a resistance phenotype that is sufficient to allow for survival long enough to reproduce. Resistance phenotypes are produced with remarkable regularity in insect populations, and their underlying mutational genotypic changes are tightly conserved, even between species [1].

The imposition of insecticides onto target and non-target insect populations presents a rapid and dramatic addition to their ecological niche. If they are to survive, their response, drawn from the variation within their genomes, must also be rapid. Two broad scenarios are proposed to explain the rapid evolution of resistance. In one, an insecticide resistance phenotype is likely to be constructed using several unrelated components if sufficiently pressured to evolve within a comparatively small number of generations. This could be necessary under conditions of intense insecticide selection where genes not normally associated with each other at the physiological level are roped together into a loose confederation. Such a confederation then becomes a collection of resistance related genotypic changes, each of independent origin, occurring timeously under conditions of insecticide selection to present as a unified system for the production of resistance. The confederation would be tightly linked under conditions of insecticide selection and could easily disassemble if selection were relaxed. Alternatively, an insecticide resistance phenotype is likely to evolve under intense selection as a single major effect controlled by one or a very small number of mutant alleles or gene duplications. The downstream physiological effect then determines the relative fitness of carrier individuals with or without insecticide selection, ultimately determining the frequency of the resistance phenotype in successive generations.

The development and increasing incidence of insecticide resistance in the major African malaria vector *Anopheles gambiae *Giles has been intensively studied over the past five decades, providing informative data on the development of resistance genotypes and phenotypes.

## *Anopheles gambiae *systematics

*Anopheles gambiae *sensu stricto is the nominal member of the *An. gambiae *species complex. Members within this complex vary widely in their behaviours and malaria vector competence, and they can be identified to species level using species specific markers including iso-enzyme alleles, cytogenetic banding sequences and non-coding DNA sequences [2-4]. *Anopheles gambiae *is widespread across tropical Sub-Saharan Africa [5], and is usually afforded the status of being Africa's most important malaria vector along with *An. funestus *Giles. However, its status as a single taxon is under revision. Cytogenetic and molecular evidence shows that *An. gambiae *is genetically structured as a set of discreet breeding units that rarely interbreed. Five chromosomal forms (Bamako, Bissau, Forest, Mopti & Savanna) and two molecular forms (M and S) are recognised [6-8]. The relationship between these two clusters of breeding units is complex and the closest associations between them are found within niche partitioning through divergent adaptation [9,10]. It is likely that the M and S molecular forms are distinct species [11-13], and there are distinct differences in the assortment of insecticide resistance genotypes and phenotypes between them.

## Detecting and characterising resistance mechanisms

Insecticide resistance phenotypes are usually assayed using response-to-exposure tests. The most widely used is the standard WHO insecticide susceptibility test for adult anophelines [14]. Using these tests, insecticide resistance phenotypes in *An. gambiae *M and S forms have been assayed from a wide array of localities across Sub-Saharan Africa. Instances of resistance to organochlorine, pyrethroid (types I and II), carbamate, organophosphate and cyclodiene insecticides have been recorded in M and S form populations [15-37].

Descriptions of the underlying resistance mechanisms and the mining of mutant alleles responsible for these physiological adjustments have proved more problematic. Several methods have been employed, in most cases led by response-to-exposure assays. Sequencing of known insecticide target site loci has identified point mutations associated with resistance [38-40]. These mutations induce amino acid substitutions leading to alterations in the structural and chemical attributes of target proteins, rendering them less susceptible to insecticide binding. Such changes in insecticide affinity can be assayed biochemically [41,42], and biochemical techniques also allow for the quantification of detoxification enzyme activities in association with insecticide resistance [43,44]. These assays are most informative when backed by quantification of the effects of specific insecticide synergists on resistance phenotype expression. Degenerate oligonucleotides designed from the genome sequences of other insect species have been used to isolate potential detoxification genes in *An. gambiae*, and subsequent RNA transcription assays have been used to quantify gene expressions in association with resistance [45]. Facilitated by the sequencing of the *An. gambiae *genome [46], gene regulation and expression of those genes associated with insecticide resistance can now be quantified by microarray and subsequent quantitative polymerase chain reaction assay (qPCR), and specialised microarrays are commercially available [27,47,48]. Lastly, genetic linkage disequilibrium analysis and the physical mapping of insecticide resistance quantitative trait loci using proximity to microsatellite markers and single nucleotide polymorphisms (SNP's) has proved extremely useful [40,49-51]. These two approaches are particularly robust because, unlike most other methods, they make no prior assumptions about the resistance mechanisms involved.

## Pyrethroid and DDT resistance

The mechanism most commonly associated with resistance to DDT and pyrethroids in *An. gambiae *is a reduced target site sensitivity termed knock down resistance (*kdr*). Two *kdr *mutations at position 1014 of the S6 transmembrane segment of the sodium channel gene have been identified. The L1014F mutation induces a leucine to phenylalanine substitution whilst the L1014 S mutation induces a substitution of the same leucine with serine [38,39]. In both cases, polymerase chain reaction (PCR) diagnostic assays have been developed allowing for the genotyping of individual mosquitoes at this locus, and the co-occurrence of both mutations in single populations has been documented [52]. However, questions over the reliability of inferring resistance phenotype based solely on the diagnosis of *kdr *genotype have been raised, because correlations between phenotype and *kdr *genotype are obscure in some instances. Recent data suggest that the correlation between response-to-insecticide phenotype and *kdr *genotype in *An. gambiae *is strongest in association with DDT, weaker in association with permethrin (type I pyrethoid) and weakest in association with deltamethrin (type II pyrethroid) [31,36,53,54]. Correlations deviating significantly from absolute imply the presence of resistance factors other than *kdr *[36,54-56] and these likely involve metabolic detoxification as has been demonstrated in *An. gambiae *populations from Kenya [50], Nigeria, Benin [27,48], Uganda [36] and Ghana [57,58].

Metabolic detoxification is the most common mode of insecticide resistance in insects [59]. In order for detoxifying enzyme systems to produce effective resistant phenotypes, transcription and enzyme production must be sufficient to catalyze the metabolism of insecticide at a rate that prevents significant interaction between the insecticide and its neuronal target. Metabolically mediated pyrethroid resistance in *An. gambiae *is most commonly based on P450 monooxygenase detoxification, with esterases implicated in a few cases. Although both of these enzyme classes are large, resistance tends to emerge in association with the upregulated activities of one or a very small number of genes [45,48,58]. There are also instances where *kdr *is not implicated in DDT resistance in *An. gambiae*. In these cases the upregulated expressions of specific glutathione-S transferases (GST's) are responsible for the metabolic conversion of DDT [60,61], although single P450 genes have also been shown to metabolise DDT [62,63]. Nevertheless, *kdr *is widespread in *An. gambiae *[64] and there is a strong trend toward increasing *kdr *frequencies in *An. gambiae *populations owing to insecticide selection pressure [65]. Further, *kdr *haplotypes have arisen independently at least four times in *An. gambiae *[66] and it is highly likely that the presence of *kdr *in the M form was transferred through introgression from the S form [67]. These data show that the *kdr *locus presents as a strong candidate for selection in the presence of DDT and type I pyrethroids.

In summary, DDT resistance in *An. gambiae *is usually conferred either by *kdr *or by GST mediated detoxification, aligning best with the development of single major effect genes. On the other hand, pyrethroid resistance is most likely to emerge as a combination of *kdr *and metabolic detoxification, aligning best with the concurrent development of several resistance factors. Micro-array analysis of a Nigerian *An. gambiae *population provides a useful example of a resistance confederation, where differential gene expression identifies several resistance associated factors including detoxification genes and cuticle deposition genes. These present in this population in conjunction with *kdr*, leading to significant pyrethroid resistance [27].

## Carbamate and organophosphate resistance

Carbamates and organophosphates share acetylcholinesterase as their target site, and at least two functional mutations in acetylcholinesterase 1 (*ace-1*) have been identified in insect species that offer reduced target sensitivity to intoxication [68]. One of these, *ace-1R *(G119S), is most commonly associated with resistance to these insecticides in *An. gambiae *[30,69,70]. This mutation is found in association with resistance in the M and S molecular forms [34], and sequence comparison between forms at this locus suggests a unique mutational event that co-occurs in both forms through introgression from the S form [71].

Esterase mediated sequestration of carbamates and organophosphates is documented for a number of insect species [72-74] and there is some evidence of this mode of resistance to the carbamate bendiocarb in *An. gambiae *S form from the Democratic Republic of Congo (unpublished data). This mode of resistance also develops as a single major effect that tends not to appear in conjunction with acetylcholinesterase target site mutations.

## Cyclodiene and phenyl pyrazole resistance

Cyclodienes and the phenyl pyrazole insecticide fipronil are antagonists of the GABA-gated chloride channel. Dieldrin resistance was first described in *An. gambiae *in Nigeria [75]. It was shown to be inherited in a simple Mendelian fashion with evidence of two resistance alleles for the same locus, one dominant and the other codominant [76-78]. Resistance to dieldrin (*rdl*) is widespread in *An. gambiae*, particularly in the West African region [79], and has been associated with mutations occurring in the M2 transmembrane domain of the γ amino-butyric acid (GABA) receptor in various insect species [80]. Cross resistance between dieldrin and fipronil has been recorded in the two *An. gambiae *laboratory strains IAN P20 and CIG [81] and a mutation conferring the substitution alanine296 to glycine is associated with dieldrin resistance in these strains [40]. Evidence of a P450 mediated metabolic component, in addition to *rdl*, has been suggested for an *An. gambiae *S form population in Ghana [79].

## Pleiotropy

Pleiotropy is used here in the classical sense as the effect of a single gene/factor on multiple traits. Pleiotropy is a direct consequence of reduced target site sensitivity mutations (*kdr*, Ace-1R and *rdl*), which not only confer reduced sensitivity to insecticide but also allow for continued ion flow regulation and enzyme function. This dual functionality also accounts for the highly conserved nature of these mutations across insect species.

The most important pleiotropic effect of insecticide resistance is reduced fitness [82]. Fitness costs are usually measured in terms of adaptive and reproductive characteristics as well as comparative measurements of resistance gene frequencies prior to and following insecticide selection. It is likely that *kdr *in *An. gambiae *carries reduced fitness in the absence of insecticide [65], although super-*kdr *in house flies appears stable [83], as does *kdr *in the peach-potato aphid [84]. There is however evidence of selection against *kdr *homozygotes in peach potato aphids in the absence of insecticide [85]. *Anopheles gambiae *individuals homozygous for *ace-1R *are likely to show enhanced fitness only in the presence of insecticide [86], because their pupal mortality is high and their body weight compromised in comparison to wild-type homozygous individuals [70]. Dieldrin resistance in association with *rdl *mutations reduces fitness in the absence of cyclodienes in *An. gambiae *and *An. stephensi *[87,88] and, to a lesser extent, in *Drosophila *[1]. In *An. gambiae*, homozygous resistant (RR) samples showed reduced fecundity in females and reduced mating competitiveness and stimulus flight response in males compared to the other genotypes [87,88].

DDT resistance by GST mediated metabolism does not incur a fitness cost in *An. sacharovi *[89], and this is likely the case for *An. gambiae *as well [90]. Similarly, P450 mediated pyrethroid resistance does not incur a fitness cost in *An. funestus *[91]. A common observation in these cases is the persistence of resistance phenotypes, in wild populations and laboratory colonies, in the absence of insecticide selection. However, a P450 pyrethroid resistance genotype associates with reduced fitness in *Culex pipiens quinquefasciatus *[92].

The effect of pleiotropy is also important at the chromosomal level. For example, dieldrin resistance in *An. gambiae *has been chromosomally mapped to division 23C on chromosome arm 2L [46,93,94]. This position falls within the breakpoints of paracentric inversion 2La, which is an extremely common inversion polymorphism in *An. gambiae*. Inversion 2La is highly stable as a polymorphism in the *An. gambiae *IAN P20 and CIG laboratory colonies [81], because both show positive heterosis whereby 2La+/2La heterozygotes are typically found in excess when compared to that expected under Hardy-Weinberg assortment. Further, dieldrin resistant and susceptible females, characterised as such by their responses to dieldrin exposure, show a close association between the "standard" arrangement 2La+ and the resistance phenotype. Individuals carrying the 2La+/2La+ and 2La+/2La arrangements were able to survive exposure to dieldrin whilst those with the alternative 2La\2La arrangement could not, with only a few exceptions in CIG [81]. These data suggest that dieldrin resistance in these two colonies is continually maintained at a high level (phenotypic frequency of approximately 75%) by the continual maintenance of inversion 2La as a polymorphism [90]. Despite the effects of a fitness cost associated with dieldrin resistance in *An. gambiae *[87,88], cross-over suppression associated with inversion polymorphism coupled with the positive heterotic effect of 2La in these colonies ensures the continual inheritance of the dieldrin resistance allele through successive generations without insecticide selection. Generally, inversion heterokaryotypes carry a fitness advantage through multiple heterozygosity at loci within the breakpoints [95], and this enhanced fitness is inadvertently conferred on the dieldrin locus by linkage disequilibrium. Inversion 2La is also associated with larval habitat [96], adaptation to aridity [97,98], resistance to desiccation [99] and *Plasmodium *infectivity [100]. These traits affect the assortment and frequencies of 2La genotypes, and are likely to exert a strong influence on the frequency of dieldrin resistance where it occurs in *An. gambiae*.

The development of multiple resistance mechanisms conferring resistance to multiple insecticides in single populations has been recorded in *An. gambiae *[19,21,101]. These scenarios are likely to have developed as a result of prolonged insecticide selection, and linkage disequilibria between their controlling loci may influence the spectrum and frequencies of within population resistance phenotypes over time, depending on the conditions of selection.

Under prolonged insecticide selection, the relative dominance or recessivity of resistance alleles defines the rate at which they are likely to approach fixation. Most reduced target site sensitivity mutations are recessive, and recessive alleles only present for selection when homozygous. As such the selection for resistance under conditions of recessivity is initially slow because most resistance allele carriers are heterozygous at affected loci. However, the complete exclusion of wild-type alleles under conditions of resistance recessivity enables a rapid subsequent increase in resistance allele frequency toward fixation. Resistance allele dominance can also lead to fixation under selection but the process tends to be prolonged because wild-type alleles survive selection in heterozygous carriers. Those factors controlling enzyme-mediated detoxification are likely to be dominant or co-dominant in expression. Whether by dominance or recessivity, resistance allele fixation can occur if insecticide selection is sufficiently intense and prolonged, and fixation at resistance loci will ultimately negate the deleterious fitness effects of resistance alleles.

The reduced fitness effects of deleterious resistance alleles can also be compensated under conditions of prolonged selection without the need for fixation. Prolonged selection allows for the development of small effect compensatory mutations whose additive phenotypic effects negate the reduced fitness associated with the major effect gene [102].

## Conclusion

Insecticide resistance mechanisms and their controlling genetic factors are generally highly conserved in insects. Despite this, the incidence of insecticide resistance is increasing in malaria vector species. In *An. gambiae*, insecticide resistance phenotypes usually develop under the control of single major genetic factors. Those factors involving mutations in target site loci are likelier to reduce fitness and are only advantageous to carriers in the presence of insecticide. Selection generally acts against these alleles and they tend to drift out of populations in the absence of insecticide. However, a combination of factors producing a single resistance phenotype also occurs in some instances. These factors invariably involve metabolic detoxification, are less likely to reduce reproductive and physiological fitness in carriers, and tend to be stable over time, even in the absence of insecticide selection. Resistance allele fixation, compensatory mutations and linkage disequilibrium - particularly that associated with polymorphic chromosomal inversions - can lend stability to otherwise deleterious resistance alleles, facilitating their continual inheritance through generations regardless of the presence or absence of selection.

Malaria vector control is becoming increasingly reliant on successfully managing insecticide resistance, which forms a crucial part of broader integrated vector management (IVM) [103]. Therefore, the characterisation of resistance mechanisms and their pleiotropic effects is important, as this information offers directives for each target vector population by identifying which control strategies are likely to prove most effective against them.

## Competing interests

The authors declare that they have no competing interests.

## Authors' contributions

BDB drafted the manuscript, LLK edited and contributed to certain sections. All authors read and approved the final manuscript.
